# Radiotherapy and High-Dose Interleukin-2: Clinical and Immunological Results of a Proof of Principle Study in Metastatic Melanoma and Renal Cell Carcinoma

**DOI:** 10.3389/fimmu.2021.778459

**Published:** 2021-10-27

**Authors:** Jenny Bulgarelli, Claudia Piccinini, Elisabetta Petracci, Elena Pancisi, Anna Maria Granato, Francesco de Rosa, Massimo Guidoboni, Massimiliano Petrini, Valentina Ancarani, Giovanni Foschi, Antonino Romeo, Luca Tontini, Ugo De Giorgi, Cristian Lolli, Giorgia Gentili, Linda Valmorri, Alice Rossi, Fabio Ferroni, Carla Casadei, Pietro Cortesi, Laura Crudi, Laura Ridolfi

**Affiliations:** ^1^ Immunotherapy, Cell Therapy and Biobank Unit, IRCCS Istituto Romagnolo per lo Studio dei Tumori (IRST) “Dino Amadori”, Meldola, Italy; ^2^ Unit of Biostatistics and Clinical Trials, IRCCS Istituto Romagnolo per lo Studio dei Tumori (IRST) “Dino Amadori”, Meldola, Italy; ^3^ Radiotherapy Unit, IRCCS Istituto Romagnolo per lo Studio dei Tumori (IRST) “Dino Amadori”, Meldola, Italy; ^4^ Department of Medical Oncology, IRCCS Istituto Romagnolo per lo Studio dei Tumori (IRST) “Dino Amadori”, Meldola, Italy; ^5^ Radiology Unit, IRCCS Istituto Romagnolo per lo Studio dei Tumori (IRST) “Dino Amadori”, Meldola, Italy; ^6^ Anesthesiology Service, IRCCS Istituto Romagnolo per lo Studio dei Tumori (IRST) “Dino Amadori”, Meldola, Italy; ^7^ Cardio-Oncology Unit, IRCCS Istituto Romagnolo per lo Studio dei Tumori (IRST) “Dino Amadori”, Meldola, Italy; ^8^ Oncology Pharmacy Unit, IRCCS Istituto Romagnolo per lo Studio dei Tumori (IRST) “Dino Amadori”, Meldola, Italy

**Keywords:** metastatic melanoma, renal cell carcinoma, radiotherapy, high dose IL-2, IFN-γ ELISPOT assay, clinical immunomonitoring

## Abstract

High-dose interleukin-2 (HD IL-2) has curative potential in metastatic melanoma (MM) and renal cell carcinoma (RCC). Radiotherapy (RT) kills cancer cells and induces immunomodulatory effects. Prospective trials exploring clinical and immunological properties of combined RT/HD IL-2 are still needed. We designed a phase II, single-arm clinical trial for patients with MM and RCC. The treatment schedule consisted of 3 daily doses of 6-12 Gy of RT to 1-5 non-index metastatic fields, before IL-2 at the first and third treatment cycle. HD IL-2 was administered by continuous infusion for 72 hours and repeated every 3 weeks for up to 4 cycles, thereafter every 4 weeks for a maximum of 2 cycles. The primary endpoint was the immunological efficacy of the combined RT/HD IL-2 treatment (assessed by IFN-γ ELISPOT). Nineteen out of 22 patients were evaluable for immunological and clinical response. Partial response occurred in 3 (15.7%) patients and stable disease was observed in 7 (36.8%). The disease control rate was 52.6% after a median follow up of 39.2 months. According to Common Terminology Criteria for Adverse Events 4.0 (CTCAE 4.0), the majority of toxicities were grade 1-2. Immunological responses were frequent and detected in 16 (84.2%) patients. Increased levels of IL-8 and IL-10 in melanoma, circulating effector memory CD4+ and intratumoral CD8+ T cells in both tumor types were detected after therapy. Overall the treatment was well tolerated and immunologically active. Immunomonitoring and correlative data on tumor and peripheral blood cell subsets suggest that this combination treatment could be a promising strategy for patients progressing after standard treatments.

## Introduction

High-dose interleukin-2 (HD IL-2) has been reported to obtain an objective clinical disease regression in 15-17% of patients with metastatic melanoma (MM) and renal cell carcinoma (RCC), with 6-8% of cases experiencing a durable complete response in all metastases (1–3). Immunotherapy is being increasingly used for the treatment of numerous cancers and has considerably changed the therapeutic scenario of MM and RCC in recent years (4). The explosion of cancer immunotherapy has raised such interest in cytokines and their role in immune stimulation that numerous studies are ongoing to evaluate new ways of targeting IL-2 receptors (5, 6). HD IL-2, one of the earliest immunotherapies, still has curative potential in a subset of patients and is an important option for those who develop side-effects or progress to previous treatments (7–9). Fyfe et al. reported findings on 255 patients with metastatic RCC undergoing HD IL-2 and enrolled in seven phase II clinical trials. The overall results showed a median duration of response of 54 months for all responders [20 months for partial responders (PRs) and not reached for complete responders (CRs)], with a median overall survival (OS) of 16 months (10). Despite its therapeutic efficacy, HD IL-2 is associated with numerous side-effects, in particular, capillary leak syndrome (CLS) from lymphoid infiltration, which has been observed histologically in many organs (11, 12). Even if most of the severe side-effects are in general reversible, the management of these adverse events may often leads to hospitalization in an Intensive Care Unit (ICU) if the medical Oncology Unit is lacking an adequate trained staff.

It is known that the administration plan for immunotherapy is a critical issue for toxicity management (14). Continuous infusion may be more beneficial than bolus dosing in terms of inducing a lower degree of cytotoxicity mediated by lymphokine-activated killer (LAK) cells and higher rebound lymphocytosis. In fact, Quan et al. administered a 72-hour high-dose continuous infusion with a more frequent schedule, observing good tolerance and activity in both MM and RCC patients (15).

Although HD IL-2 remains an important option in the curative treatment strategy of MM and RCC, there is clearly a need to optimize sequencing and to find potential new therapeutic combinations to improve its therapeutic index. Radiotherapy (RT), an important component of cancer treatment, is highly effective at directly killing cancer cells and surrounding cells within the tumor stroma. When a tumor is irradiated, cellular stress or injury within the tumor may lead to the release of antigens (i.e., tumor-associated antigens (TAAs), eliciting immunogenic cell death and resulting in the release of cytokines and damage-associated molecular patterns (DAMPs) that trigger innate immunity signalling pathways. These signals favor the recruitment of antigen presenting cells (APCs) such as dendritic cells (DCs), promote uptake of dying tumor cells, and enhance TAA processing and the cross-presentation of antigenic peptides *via* major histocompatibility complex (MHC) class I to CD8+ T cells, promoting an adaptive immune response (16). Sometimes these immunomodulatory effects has been observed also in the tumor microenvironment (TME) of micro- and macrometastases far away from the irradiation site (*Abscopal effect*) and mediate the systemic immune response. Together with the potential to initiate new immune responses, radiation therapy is influenced also by the pre-existing immunity at the tumor site, where T cells form a resident memory population that is associated with responsiveness to treatment (17). The delivered dose of RT also affects the anti-tumor response: a dose >2 Gy induces higher tumor antigens spreading and has been described to be associated with the generation of effective and durable CD8^+^ T cell-mediated immune response, resulting in local and distal tumor control (18). Such evidence provides a strong rationale for combining RT and immunotherapy. In fact, numerous clinical trials have confirmed the booster effect of RT combined with immunotherapy in several tumor types, including MM and RCC, and are in the process of exploring the association between different schedules and dosage of RT and immune checkpoint inhibitors (ICIs) (19, 20).

Two active areas of investigation in the field of immunotherapy focus on the elimination of immune suppression/regulation and on improved patient selection. The IFN-γ ELISPOT assay monitors both cytotoxic T lymphocyte (CTL) frequency and function and has been widely used in multiple cancer immunotherapy trials to analyze immune response before and after each treatment cycle (21–23). This functional test is also used in patients with MM or RCC undergoing IL-2 based immunotherapy to detect specific immune responses against selected tumor antigens and could represent a reliable biomarker of response to treatment (24, 25). At the same time, considering the treatment’s immune modulation-based mechanism of action, the analysis of the tumor microenvironment (TME) and the frequency and proportion of the circulating T cell population and peripheral cytokine profiling are all elements that can potentially help to identify the patients who are more likely to benefit from treatment (26–28).

We thus designed a single-arm phase II trial to evaluate: *i)* the immune response after sequential treatment with RT and HD-IL-2 in patients with MM and RCC; *ii)* the safety of the treatment combination, the clinical response rate and the OS; *iii)* the role of immune biomarkers in predicting clinical response and prognosis.

## Materials and Methods

### Study Design and Participants

This was a Phase II proof-of-principle, single-center, open-label, single-arm clinical trial (ClinicalTrials.gov NCT01884961; EudraCT no. 2012-001786-32). Patients with MM or RCC with a measurable disease progressed after at least 1 line of therapy, life expectancy ≥3 months and with tissue sample availability were considered eligible and included in the study (**Table 1** and **Supplementary Table S1**). Patients with not treated or uncontrolled brain metastases were excluded [Detailed inclusion and exclusion criteria are described in ref (29).]. The study was approved by the CEIIAV Ethics Committee (approval n° 2217.2012.I.5.81 of 13/06/2012) and was conducted in accordance with the principles laid down in the 1964 Declaration of Helsinki. Written informed consent was obtained from all participants. The study had a Simon’s minimax two‐stage design, with 7 patients planned for stage one (30). If three or more immunological responses were seen in stage one, an additional 12 patients would be accrued in stage two for a total of 19. If fewer than three immunological responses were seen, the study would be terminated. This was based on a defined unacceptable immunological response rate of 40% and acceptable rate of 70%, with 10% type I error and 90% power. At the end of the study, if 11 immunological responses or more were observed, the treatment could be considered effective.

**Table 1:** Patient characteristics.

Tumor type	RCC (n = 9)	MM (n = 10)	Total (n = 19)
**Gender**			
Female, n (%)	3 (33.3)	6 (60.0)	9 (47.4)
Male, n (%)	6 (66.7)	4 (40.0)	10 (52.6)
**Age at start of treatment, years**			
Median [min - max], IQR	60.8 [38.0 – 71.9], 8.7	55.4 [35.3 – 71.8], 25.4	60.8 [35.3 – 71.9], 25.0
ECOG PS*, n (%)			
0	6 (85.7)	9 (100.0)	15 (93.8)
1	1 (14.3)	0 (0.0)	1 (6.2)
**Number of metastatic sites, n (%)**			
≤2	2 (22.2)	5 (50.0)	7 (36.8)
>2	7 (77.8)	5 (50.0)	12 (63.2)
**Number of previous lines of therapy, n (%)**			
0	1 (11.1)	–	1 (5.3)
1	1 (11.1)	1 (10.0)	2 (10.5)
2	1 (11.1)	3 (30.0)	4 (21.0)
≥3	6 (66.7)	6 (60.0)	12 (63.2)

RCC, renal cell carcinoma; MM, metastatic melanoma; IQR, interquartile range; ECOG, Eastern Cooperative Oncology Group; PS, performance status.

*The total does not add up to the total due to a missing value.

### Treatment

**Figure 1:**
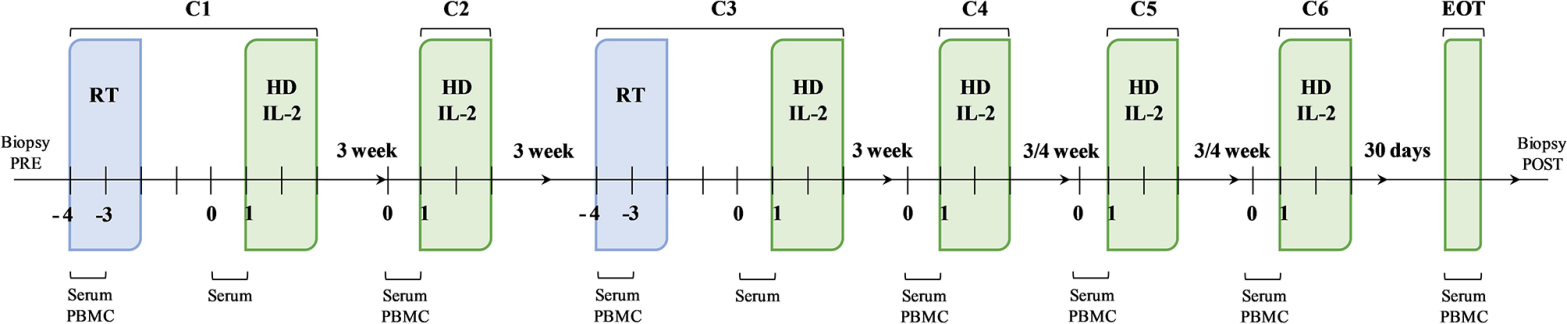
Schematic representation of the therapeutic schedule and biological sample collection. The therapeutic strategy was based on a sequence of therapeutic cycles performed every 3 weeks up to cycle 4 (C4) and every 4 weeks thereafter for a further 2 cycles, up to a maximum of 6 months of treatment. At the first (C1) and third (C3) cycle, the administration of high-dose interleukin-2 (HD IL-2) was preceded by radiotherapy. The second (C2), the fourth (C4), the fifth (C5) and the sixth (C6) cycles consisted in the administration of HD IL-2 only. Biological samples were collected at established times during the treatment.

After eligibility assessment, enrolled patients received RT and HD IL-2. In particular, the treatment schedule consisted of 3 daily doses of 6-12 Gy of RT in 1 to 5 non-index metastatic fields administered before IL-2 at the first and third treatment cycle. The lesions to be irradiated were selected upon clinical judgement. In particular, priority was given to symptomatic lesions distant to previously irradiated sites and treatable with no dose concerns for the surrounding critical organs, avoiding lesions undergoing study biopsy. Moreover, only 1 tumor site was irradiated in each RT cycle for each patient. HD IL-2 (Proleukin, 18 MIU/m2 per day in 500 cc) was administered after RT by continuous infusion over 72 hours and every 3 weeks thereafter for up to 4 cycles, then every 4 weeks for a further 2 cycles up to a maximum of 6 months of treatment (**Figure 1**). During the three-day IL-2 infusion, dexamethasone (4 mg twice/day), ondansetron, paracetamol and intravenous hydration were administered to patients. We chose to use corticosteroids during the 3 days of IL-2 infusion to make the therapy more manageable confident that the use of low-dose steroids for very short times and with the intent to counteract a toxicity (and not a symptomatic disease) would not have affected the therapy according to previously reported studies (31–34). Before starting the treatment, a peripherally central catheter (PICC) for HD IL-2 infusion and a peripheral venous access for hydration were inserted. All patients were hospitalized in the Medical Oncology Ward of our institute and were monitored for vital signs every 6 hours (29).

### Assessment of Clinical Response and Adverse Effects

Radiological evaluations performed by computed tomography (CT) or magnetic resonance imaging (MRI) were planned every 2 cycles of treatment. Soft tissue response, stability and progression in images were assessed according to RECIST version 1.1 (RECIST 1.1) guidelines. iRECIST (Response Evaluation Criteria in Solid Tumors in immunotherapy trials) criteria were used to evaluate tumor response, with a reassessment after 4 weeks to confirm or not suspected disease progression (35, 36). Toxicity assessment was based on CTCAE 4.0. Delays in IL-2 dose were allowed within the same cycle but no dose reductions were permitted. A delay longer than 24 hours resulted in discontinuation of that cycle of IL-2.

### Biological Sample Collection

Peripheral blood mononuclear cells (PBMCs) and serum samples were longitudinally collected at the first and third treatment cycles before and after RT and before every IL-2 infusion at the second, fourth, fifth and sixth treatment cycle. Further PBMC and serum samples were collected after the end of treatment. Tumor biopsies were also performed before and after treatment for immunohistochemistry (IHC) analysis (**Figure 1**). Clinical data (white blood cell count, absolute lymphocyte, neutrophil, monocyte and platelet counts) were collected for all patients before starting treatment and at the end of the treatment.

### INF-γ ELISPOT Assay

The immunological efficacy of the treatment was evaluated by the IFN-γ ELISPOT assay kit (U-CyTech biosciences, Utrecht, The Netherlands) on thawed PBMC samples resuspended in RPMI 1640 medium to assess circulating effector cells activated by selected TAAs and secreting IFN-γ. In particular, we used overlapping peptides representing full-length sequences (peptide pool) of Melan-A, PMEL and tyrosinase (JPT Technologies, Berlin, Germany) for MM; 5T4, CA IX and EGFR (Mimotopes Pty Ltd, Mulgrave, Australia) for RCC; and MAGE-A3, NY-ESO1 and survivin (JPT Technologies) for both tumor types.

Briefly, 96-well plates equipped with PVDF membranes were incubated with ethanol 70% for 2 minutes at room temperature, after which coating antibody against IFN-γ was added to each well and incubation continued for one hour in the dark at 37°C. Blocking solution was then added and the plates were incubated overnight in the dark at 4°C. Each peptide pool and CEF control peptides (JPT Technologies) were reconstituted with DMSO and diluted with RPMI 1640 medium at a concentration of 1μg/mL. PBMCs (1×10^5^ cells/well) and the peptide pool were added to each well and incubated overnight in the dark at 37°C. Each sample was tested in quadruplicate and CEF control peptides were used as a positive control of the test. Cells were left untreated in RPMI 1640 medium as negative control. Wells were supplemented with the secondary biotinylated and incubated in the dark at 37°C for one hour. HRP-conjugated streptavidin was added and incubation continued in the dark at 37°C for one hour. Finally, the substrate was diluted in ethanol 30% and added to each well followed by incubation for 25 minutes at room temperature. Each step was followed by a washing stage with PBS 1X. PVDF membranes were dried in the dark at room temperature. The plates were evaluated using the A.EL.VIS ELISPOT Reader and A.El.VIS GmbH V 5.1 software (Thema Ricerca, Bologna, Italy). The software detects the secretion of IFN-γ as a spot of red substrate precipitated by each activated effector cell.

### Immunohistochemistry

IHC staining was performed on 4-μm-thick formalin-fixed paraffin-embedded (FFPE) pre- and post-treatment tissue sections from tumor biopsies to evaluate the level of TAAs specifically expressed in MM (Melan-A, PMEL, tyrosinase), RCC (EGFR, CAIX) and both tumor types (MAGE-A3, survivin, NY-ESO1). The staining was performed using the Ventana Benchmark XT automatized system (Ventana-Roche, Oro Valley, Arizona, USA). OptiView DAB IHC Detection Kit was used to detect the TAAs of RCC samples, while UltraView AP Fast Red was used for MM, as per the manufacturer’s instructions. The EGFR expression level was assessed in RCC tumor biopsies by EGFR pharmDX manual kit (Cat# K1492 monoclonal mouse IgG1, clone 2-18C9, Agilent technologies, Santa Clara, CA, USA) in accordance with the manufacturer’s recommendations. After deparaffinization, rehydratation in graded ethanol and antigen retrieval, the slides were incubated with the primary antibodies, as described in **Supplementary Table S2**. Slides were counterstained with Carazzi hematoxylin and evaluated under a light microscope. Semiquantitative analysis was performed by evaluating the percentage of positive cells and assessing the staining intensity with a score ranging from 0 to 3 on five representative fields. All the analyses were checked by a senior pathologist (MG).

If additional sections of the same biopsies were available, the presence of intratumoral T cells were also assessed with anti-human CD3, CD8, FOXP3 and granzyme β antibodies, as reported in **Supplementary Table S2**. Staining was performed using the OptiView DAB IHC Detection Kit. For FOXP3 and GZMB detection in melanotic MM and in some RCC matched biopsies, a non-biotin Poly HRP conjugate system followed by 3-amino-9-ethylcarbazole (AEC) substrate reaction was used instead of 3,3’-diaminobenzidine (DAB), as previously described (37). Slides were counterstained with Carazzi hematoxylin. High-resolution whole slide images (WSI) (40× and 20× magnifications) of IHC stained slides were acquired using the Aperio CS2 slide scanner (Leica Biosystems, Nußloch, Germany) or the MicroVisioneer Manual WSI system (MicroVisioneer, Esslingen am Neckar, Germany, 20× magnification). A quantitative analysis was performed with Qupath software and checked by a senior pathologist (MG) to study the immune infiltrate (37).

### Flow Cytometry

Phenotypic analysis of the circulating lymphocyte subpopulation was performed by multiparametric flow cytometry. Briefly, PBMCs longitudinally collected before, during and after treatment were thawed and stained at 4°C in the dark for 30 minutes with the following anti-human monoclonal antibodies or respective isotype controls: anti CD3 PercP-Cy 5.5 clone: OKT3 Cat#317336, anti CD45RA APC-Cy7 clone: HI100 Cat#304128 (Biolegend, San Diego, USA), anti CD8 FITC clone: HIT8a Cat#555634, anti CD4 FITC clone: RPA-T4 Cat#555346, anti CCR7 PE clone: 150503 Cat#560765, anti CD25 APC-Cy7 clone:M-A251 Cat#557753 (BD Biosciences, San Jose, CA, USA) and anti Foxp3 APC clone: 3G3 Cat#130-093-013 (Miltenyi Biotec, Bergisch Gladbach, Germany). Stained samples were acquired with FACSCanto flow cytometer (Becton Dickinson, Franklin Lakes, New Jersey, USA) and data were analyzed by Diva software (Becton Dickinson) or FlowJo Software (Tree Star, Ashland, OR, USA).

### Multiplex Cytokine Immunoassay

Serum levels of inflammatory cytokines IL-1β, IL-6, IL-8, IL-10, TNFα and IL-12p70 were determined simultaneously by a customized human 6-plex cytokine chemiluminescent Protein Array (Ciraplex) (Aushon Biosystems, Billerica, MA, USA), a multiplex array set up in 96-well plates in which each well was coated with 6 different monoclonal antibodies. Briefly, a sandwich Enzyme-Linked Immunosorbent Assay (ELISA) was performed on serum samples, as per the manufacturer’s instructions. The concentration of the analytes was measured using a chemiluminescent method and a linear regression model was used to collect and analyze the data. In addition, serum levels of VEGF and fibronectin were evaluated using the Quantikine^®^ ELISA Human VEGF kit and the Quantikine^®^ ELISA Human Fibronectin kit (R&D systems™ - Bio-Techne, Minneapolis, MN, USA), following the manufacturer’s instructions. The evaluation of optical density (OD) for each well was performed at 450 nm and 550 nm wavelengths using a spectrophotometer. The absorbance was directly proportional to the concentration of the analyte, measured using the linear regression model. Each sample was tested in duplicate and analyte concentrations were reported in pg/mL.

### Statistical Analysis

The primary endpoint of the study was immunological response, defined as an ≥10% enhancement of spot-forming cells (SFCs), measured by IFN-γ ELISPOT assay, in at least one antigen post-treatment with respect to its value before treatment.

The disease control rate (DCR) was defined as the percentage of patients with a complete (CR) or partial response (PR) or with stable disease (SD) out of the total number of evaluable patients receiving at least one treatment cycle. Progression‐free survival (PFS) was defined as the time from the start of treatment to the first radiographic evidence of progression or death, whichever came first. OS was defined as time from the start of treatment to death or last follow‐up. Data were summarized using mean ± standard error of the mean (SEM) or median and minimum and maximum values or interquartile range (IQR), as appropriate, for continuous variables, and natural frequencies and percentages for categorical ones.

Event‐time distributions were analyzed using the Kaplan-Meier method. Median PFS and OS were reported, as were the corresponding 95% confidence intervals (CIs). The median follow-up time was computed by the reverse Kaplan-Meier approach and reported together with 95% CIs. The Mann Whitney U test and Chi-square or Fisher’s exact test, as appropriate, were used to compare the two independent groups, i.e. MM and RCC. The paired Student t-test or the Wilcoxon signed rank test was used, as appropriate, to compare pre- and post-treatment values. An analysis of variance (ANOVA) for repeated measures, including an interaction term between time and tumor type, was used to explore whether potential changes over time differed on the basis of tumor type. Adjustment for potential confounding factors was performed, when appropriate. Logistic and Cox proportional hazards regression models were used to assess the association between biomarkers measured at baseline and treatment response or prognostic outcomes such as PFS or OS. Cox models with time-dependent covariates were fitted to investigate whether biomarker changes over time might affect survival. A two-sided *p*-value <0.05 was considered statistically significant. All of the analyses were carried out with STATA 15.0 (College Station, Texas, USA), R version 3.4.0 statistical software (http://cran.r-project.org) and GraphPad Prism (version 6, La Jolla, CA, USA).

## Results

### Patients

Between June 2012 and August 2017, a total of 22 eligible patients were enrolled in the study. Among these, 2 had inadequate biological samples for immunological test assessment and one patient died shortly after enrollment, precluding the possibility of assessing the primary endpoint. Thus, a total of 19 patients (10 with MM and 9 with RCC) were considered in the analysis. Baseline patient characteristics are summarized in **Table 1** and **Supplementary Table S1**. Among the patients with MM, 7 (70.0%) had uveal melanoma, 1 (10.0%) mucosal melanoma and 2 (20.0%) cutaneous melanoma. Median age was 60 years (min-max: 35-72), and 52.6% of patients were males. All MM patients harbored a BRAF and NRAS wild-type tumor and received a median of 3 lines of prior therapies including ICIs, whereas the majority of patients with RCC received at least 3 lines of tyrosine kinase inhibitors (TKIs). Only one RCC patient received this treatment as first line therapy because refused all the other proposed treatments. The median IL-2 dosage was 33 MIU/day and the most frequent sites for booster RT were lymph nodes and liver metastases. Overall, of the 19 patients evaluable for clinical response, 3 had PR (2 RCC and 1 MM), 7 showed SD (3 RCC and 4 MM) and 9 had progressive disease (PD) (4 RCC and 5 MM). The DCR was 52.6% and the median follow-up was 39.2 months. The median PFS was 3.2 months (95% CI: 1.3-14.8) for RCC and 2.9 months (95% CI: 1.3-5.4) for MM, while the median OS was 8.7 months (95% CI: 1.4-NR) and 6.7 months (95% CI: 1.9-11.9), respectively. We did not investigate whether radiated sites or volumes influenced treatment response as the study cohort is too small to answer the question, although we did not have the perception that this occurred (details regarding the volumes and the site of the radiated lesions are reported in the **Supplementary Table 1**).

### Immunological Response

**Figure 2:**
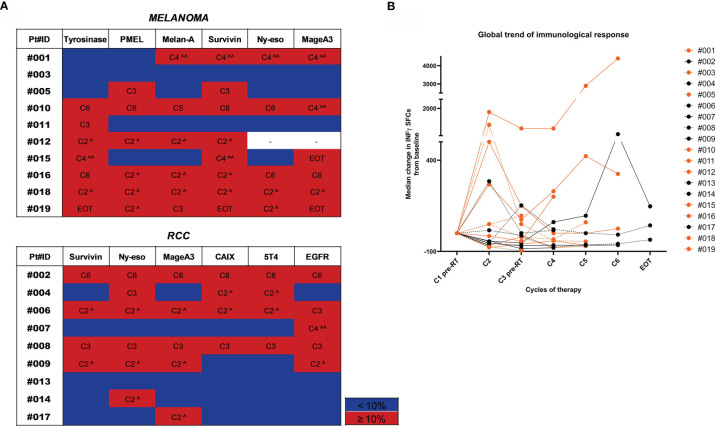
Trend of immunological response to treatment. **(A)** Heatmaps highlighting the gradient of the immunological response to treatment represented as % of variation in the number of INF-γ SFCs for each TAA compared to baseline values. Patients are divided by tumor type (MM upper panel and RCC lower panel). For each tested TAA, immune responses ≥10% compared to baseline values are highlighted in red and the therapy cycle for which the best response was observed is reported. Immune responses <10% are shown in blue. (^ after the first RT cycle, ^^ after the second RT cycle). **(B)** The overall trend of the immunological response for each patient is shown in a spider plot (orange dots/line for MM and black dots/lines for RCC). Each dot represents the median variation in the number of INF-γ SFCs for each TAA, considering the baseline value as 0. Treatment cycles are indicated in the x axis.

To capture high-magnitude T-cell response induced by HD IL-2 plus RT, we performed *ex vivo* IFN-γ ELISPOT on PBMCs incubated with TAA peptide pools. T cell responses against tumors were defined as a positive response when the number of spots after therapy was ≥10% with respect to the baseline value. Treatment-induced immunological response was frequent and detected in 84.2% (16/19) of patients in whom it was possible to measure an immunological response against at least one TAA after treatment (**Figure 2A**). The median value of the variation in immunological response against each antigen with respect to the baseline response was calculated at each therapy cycle. The trajectory over time of these median values for each patient is shown in **Figure 2B**. A >10% variation was observed after the first cycle of therapy in about half of the cases. Indeed, we detected an increase in the immunological response after the 1st cycle of the combined therapy (C2) in 9/16 patients and after the 3rd treatment cycle (C4) in 4/16 patients. In the latter group, patients #007 (RCC) and #015 (MM) showed a concomitant PR to treatment. Patient #002 (RCC) showed an immunological response against all the tested antigens after the 5th treatment cycle (C6) and a PR. The remaining 3 patients developed an immunological response after the 2nd cycle of therapy (C3). Conversely, patient #003 (MM) who progressed and patient #013 (RCC) who obtained SD did not show any detectable immunological response against the tested antigens during the course of treatment with respect to baseline levels. Overall, we detected immunological responses against all the tested antigens, the most immunogenic being EGFR, survivin and NY-ESO1 in RCC and tyrosinase, Melan-A, survivin and MAGE-A3 in MM (**Figure 2B**).

### Treatment-Related Toxicity

Adverse events and immune-related toxicities were evaluated in patients who received HD IL-2 treatment in our Medical Oncology Ward. Of note, none of the patients had to be transferred to an ICU. Fever was the most common toxicity and was easily managed with paracetamol or indomethacin infusion. Other commonly detected toxicities (grade 1-2 according to CTCAE 4.0) were erythema/rash, gastric pain, pruritus and cough. Overall, 8 patients had ≥grade 3 adverse events. No treatment-related deaths occurred (**Supplementary Table S3**). The IL-2 dose was never reduced or the infusion interrupted for >24 hours. Moreover, when IL-2 infusion followed RT treatment (cycles 1 and 3), no additional toxicities were observed.

### Modulation of Cytokine and Proangiogenic Factors by HD IL-2 Plus RT

**Figure 3:**
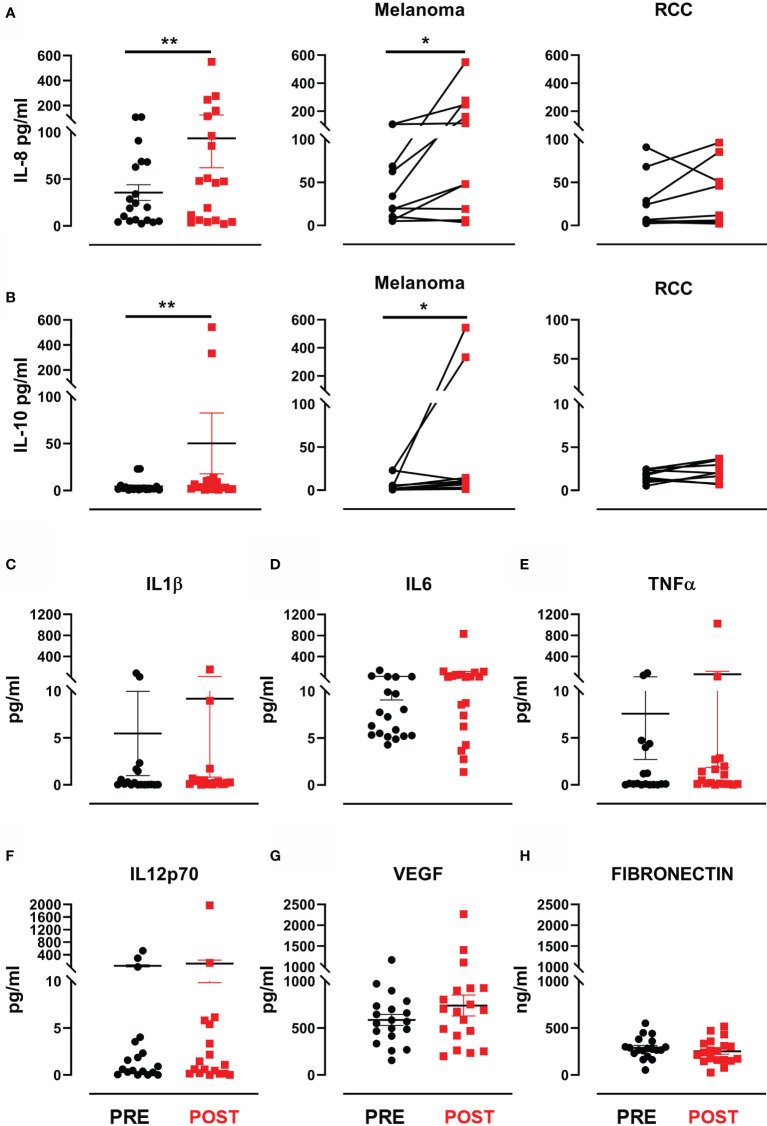
Cytokines and proangiogenic factor modulation after treatment. **(A)** Dot plot with bar represents IL-8 concentration (pg/mL) in the entire patient cohort (left panel), in MM patients (middle panel) and in RCC patients (right panel). **(B)** Dot plot with bar represents IL-10 concentration (pg/mL) in the entire patient cohort (left panel), in MM patients (middle panel) and in RCC patients (right panel). Dot plots with bar represent pre- and post-therapy concentration levels (pg/mL) of **(C)** IL-1β, **(D)** IL-6, **(E)** TNF-α, **(F)** IL-12 p70, **(G)** VEGF and **(H)** fibronectin in ng/mL. Data represent individual values, mean (central bar) ± SEM (upper and lower bar). The black circular dots represent pre- therapy samples, while the red squares represent post-therapy samples. Statistical analysis was performed with the nonparametric Wilcoxon signed rank test; *p value < 0.05, **p value < 0.01.

We looked for predictive modulations of response to treatment by evaluating serum levels of VEGF and fibronectin, known to have a prognostic value in MM and RCC, and pro-inflammatory cytokines (IL-1β, IL-6, IL-8, IL-10, TNFα, IL-12 p70) known for their involvement in local and systemic immune response. A significant increase in IL-8 levels was observed after therapy in the entire cohort (mean ± SEM 35.5 ± 8.4 (pre-treatment) *vs*. 93.6 ± 31.4 (post-treatment) IL-8 pg/mL, *p*=0.006) (**Figure 3A**). Similarly, we observed a significant increase in IL-10 levels after therapy (mean ± SEM 4.3 ± 1.5 (pre-treatment) *vs*. 50.2 ± 32.4 (post-treatment) IL-10 pg/mL, *p*=0.007) (**Figure 3B**). Such variation in both cytokines would appear to be mainly due to consistent increases in the MM subgroup (*p*=0.014 for IL-8 and *p*=0.048 for IL-10) (**Figures 3A, B**). Conversely, the levels of VEGF, fibronectin and the other cytokines did not significantly change after treatment (**Figures 3C–H**). None of the evaluated biomarkers were found to correlate with clinical outcome or predict response to treatment.

### Modulation of Peripheral Blood Cell Biomarkers by Treatment

**Figure 4:**
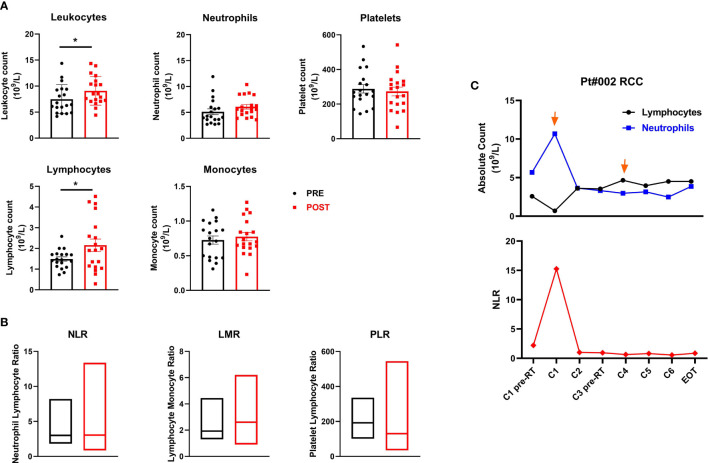
Peripheral blood cell biomarkers of immune modulation. **(A)** Scatter plots with bars represent leukocyte, neutrophil, platelet, lymphocyte and monocyte counts (10^9^/L) in peripheral blood collected before and after therapy. Data represent individual values, mean (central bar) ± SEM (upper and lower bar). The black circular dots represent pre- therapy samples, while the red squares represent post-therapy samples. **(B)** Boxes with floating bars represent neutrophil-to-lymphocyte ratio (NLR), lymphocyte-to-monocyte ratio (LMR) and platelet-to-lymphocyte ratio (PLR). The black and red boxes represent pre- and post-therapy values, respectively. **(C)** Trend of the absolute count of lymphocytes and neutrophils (upper panel) and the NLR (lower panel) during treatment cycles for patient #002 (RCC, partial responder). The black line and dots refer to the lymphocyte count, the blue line and dots to the neutrophil count and the red line and dots to the NLR. Statistical analysis was performed with the nonparametric Wilcoxon signed rank test; *p value < 0.05.

We evaluated baseline and post-treatment values of peripheral blood components, observing a significant increase in leukocyte levels (mean ± SEM 7.5 ± 0.6 *vs*. 9.1 ± 0.6 leukocyte count 10^9^/L in pre-treatment and post-treatment samples, respectively; *p*=0.014) and absolute lymphocyte count after therapy (mean ± SEM 1.5 ± 0.1 *vs*. 2.2 ± 0.3 lymphocyte count 109/L in pre-treatment and post-treatment samples, respectively; *p*=0.012). Changes were observed in absolute neutrophil, monocyte and platelet counts and in the neutrophil-to-lymphocyte (NLR), lymphocyte-to-monocyte (LMR) and platelet-to-lymphocyte (PLR) ratios when pre- and post-therapy samples were compared, although none were significant (**Figures 4A, B**). Moreover, no significant associations were found between these peripheral inflammatory biomarkers and clinical outcome. Of note, patient #002 (RCC) showed an immunological response to the treatment and a clinical PR. Studying the patient’s lymphocyte trend, neutrophil count and relative NLR in the bloodstream, we observed an initial increase in the neutrophil count after the 1st cycle of therapy followed by a stable phase and finally a decrease after the 4th treatment cycle. Conversely, the NLR decreased substantially after the 1st cycle of therapy, remaining essentially unchanged until the end of treatment (**Figure 4C**).

### The Frequency of Circulating T Cell Subpopulations Significantly Changed After Treatment

We studied peripheral CD4+ and CD8+ T cell subsets using multiparametric flow cytometry to investigate in depth the overall immunological effects of the HD IL-2 and RT combination.

**Figure 5:**
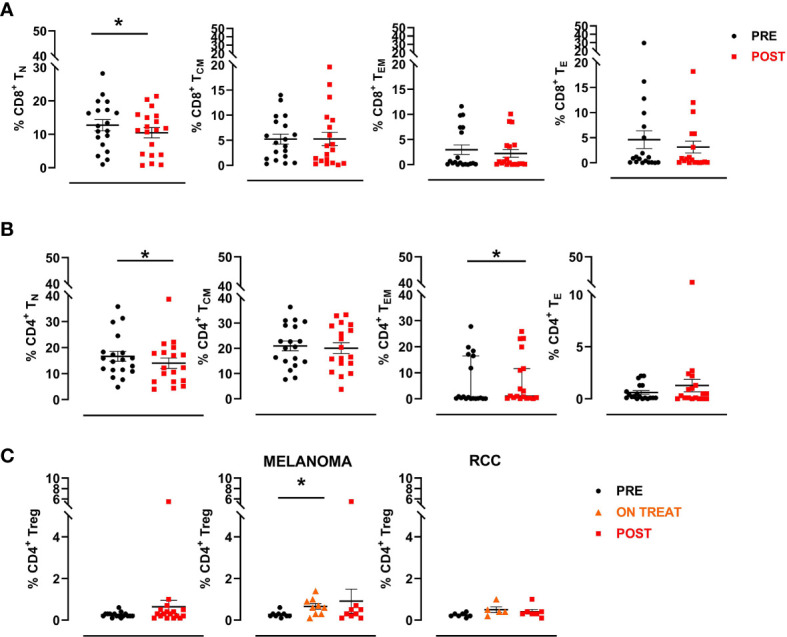
CD8+ and CD4+ T cell subsets. A manual gating strategy was used to define T cell subsets within CD8+ and CD4+ lymphocytes. Briefly, naïve T cells were identified as CCR7+CD45RA+ (TN), central memory as CCR7+CD45RA- (TCM), effector memory as CCR7-CD45RA- (TEM) and effector as CCR7-CD45RA+ (TE). **(A)** Percentages of different CD8+ T cell subpopulations in pre- and post-treatment patient samples (n=19). **(B)** Percentages of different CD4+ T cell subpopulations in pre- and post-therapy patient samples (n=19). The black circular dots represent pre- therapy samples and the red squares represent post-therapy samples. **(C)** Scatter plots representing the percentage of Treg cells (CD3+CD4+CD25highFOXP3+) in the entire patient cohort (left panel), in MM patients (middle panel) and in RCC patients (right panel). The black circular dots represent pre-therapy samples, the orange triangles represent on-treatment samples (C3 or C4), the red squares represent post-therapy samples. Data represent individual values, mean (central bar) ± SEM (upper and lower bar). Statistical analysis was performed with the nonparametric Wilcoxon signed rank test; *p value < 0.05.

Briefly, a first gate was set on physical parameters (FSC and SSC), after which viable lymphocytes were selected and a second gate was set on CD3+ CD8+ cells. Naive CD8+ T cells were then identified as CCR7+CD45RA+ (CD8+ TN), central memory as CCR7+CD45RA- (CD8+ TCM), effector memory as CCR7-CD45RA- (CD8+ TEM) and effector as CCR7-CD45RA+ (CD8+ TE). The same was done to define the different CD3+ CD4+ T cell subsets. A significant decrease in the percentage of CD8+ naïve T cells was observed after treatment (mean ± SEM 12.7 ± 1.7 *vs*. 10.5 ± 1.5 in pre-treatment and post-treatment samples, respectively; *p*=0.025), followed by a reduction, albeit not significant, in the other subpopulations with the exception of CD8+ effector memory T cells, which remained stable (**Figure 5A**). We also observed a significant decrease in the percentage of CD4+ naïve T cells (mean ± SEM 16.7 ± 1.9 *vs*.14.0 ± 2.0 in pre- and post-treatment samples, respectively; *p*=0.039) and a concurrent increase, albeit not significant, in the frequency of the CD4+ effector and effector memory subsets after therapy (**Figure 5B**). Finally, we investigated changes in the CD4+ regulatory T cell subset (Treg), defined as CD3+CD4+CD25highFOXP3+ cells, observing no significant modulation after therapy in any patient. Conversely, when we analyzed data by tumor type, we observed an increase of this cell subsets during the treatment (ON TREAT- considering for all patients C3 or C4 cycle of therapy as the point of major variation) that was significant in melanoma patients (mean ± SEM 0.3 ± 0.05 *vs*. 0.7 ± 0.1 in pre-treatment and on-treatment samples, respectively; *p*=0.016) and returned to baseline values after therapy (POST) (**Figure 5C**). Overall, no significant associations were found between T cell subset changes and clinical outcome.

### Maintenance of TAA Expression After Combined Treatment and Concurrent Increase in Intratumoral CD8+ T Cells

Tumor biopsies collected before and after treatment were evaluated for the expression of a panel of TAAs known to be highly expressed in >80% of patients with MM and RCC and used to assess the immunological efficacy of the treatment by IFN-γ ELISPOT assay. A semiquantitative evaluation was performed by 2 different operators and checked by a senior expert pathologist (MG), considering the median percentage of positive cells in five representative fields. Overall, there was no significant change in TAA expression after treatment, with some antigens already highly expressed in tumor tissue before the start of the therapy. MAGEA3 was expressed in all pre-therapy samples analyzed (16), while CAIX was expressed in all pre- (9) and post-therapy (4) RCC samples (**Supplementary Figures S1A, B**).

To gain further insight into the intratumoral T cell landscape of HD IL-2 and RT-treated patients, CD8-positive cells were quantified in five matched pre- and post- FFPE tumor tissue samples. Overall, the amount of intratumoral CD8+ T cells increased, albeit not significantly, in post-treatment samples with respect to pre-treatment samples (mean ± SEM 65.8 ± 28.9 *vs*. 239.4 ± 66.6 CD8+ cells/mm2 in pre-treatment and post-treatment biopsies, respectively; *p*=0.063). No significant changes were observed in the overall number of intratumoral CD3+ T cells, FOXP3+ cells or GZMB+ cells after HD IL-2 and RT (**Supplementary Figure S1C**). Given the shortage of tumor material, only 3 matched pre- and post-treatment tumor tissue samples were evaluated for the presence of intratumoral GZMB+ cells. Similarly, no significant changes were found in the number of CD3+, CD8+ and FOXP3+ cells in the pre-therapy biopsies of responders (3 PR and 2 SD) and non-responders (6 PD) (**Supplementary Figure S1D**).

## Discussion

Early trials of HD IL-2 showed that this treatment elicited durable responses in 10-15% of patients with melanoma and in 15-25% of those with RCC (1–3). Contemporary studies continue to report durable responses and survival rates, mainly in patients obtaining CR (38–40). Encouraged by these recent reports, the interest in IL-2 as a combination or sequential therapy to further improve objective response remains high (20, 41, 42). However, standard HD IL-2 administration usually requires hospitalization and a dedicated medical team to monitor for capillary leak syndrome and other treatment-related toxicities. Moreover, the interaction between HD IL-2 and other immunomodulating therapies such as RT are still poorly understood, indicating the need for clinical trials to identify the best sequential or combination regimen. In this respect, immune monitoring to explore immunological efficacy has proven fundamental not only for the discovery of mechanisms of response and resistance to treatment, but also for the design of combination regimens to enhance anti-tumor immunity and clinical response.

We designed the present study to evaluate the manageability and tolerability of a therapeutic schedule consisting of a short HD IL-2 infusion repeated every 21 days, corticosteroids to reduce the risk of capillary leak syndrome, and RT to exploit the synergistic effect. Our study was not designed to demonstrate an abscopal effect of RT which indeed is a very rare event to observe, but to evaluate the clinical and the immunological activity of radiotherapy + HD IL-2 and predictive biomarkers of response, speculating that radiotherapy could act as an immunological boost for IL-2 (**Figure 1**). In line with this, Curti et al. recently published the results from a prospective phase II randomized study in which stereotactic body RT followed by HD IL-2 induced a higher number of objective responses and obtained a higher DCR than IL-2 monotherapy in 44 evaluable MM patients (43). Notably, the Curti’s study population was mostly composed by treatment naive patients and the efficacy reported data are partially comparable with the ones described in our study. Nevertheless, the strong synergistic activity between IL-2 and RT described in their study remains very interesting.

To the best of our knowledge, our prospective phase II trial represents one of the few clinical studies with a unique treatment schedule aimed at reducing IL-2 toxicities and side-effects and exploring the immunomodulating properties of combined high-dose IL-2 and RT. The limitations of this trial include small sample size and the fact that it was a single-arm, non-randomized study. Notably, here we presented the results of a single-center study which, for ethical reasons, provided for the enrollment of patients progressing to approved standard therapies. These aspects have hampered the sample size of the enrolled patients cohort and the overall enrolling time. Although patients were all heavily pre-treated, we observed 3 PR and 7 SD. Interestingly, 70% of melanoma patients had metastatic uveal melanoma, which has a particularly dismal prognosis and all but two RCC patients were treated with everolimus, which is a quite strong immunosuppressive drug. Everolimus may have affected our results as it seemed to affect the efficacy of the second-line nivolumab in the Italian cohort of nivolumab Expanded Access Programme (EAP), where patients previously treated with everolimus showed just a trend of inferior OS (HR 1.30, CI0.95–1.76; P = 0.10) (44).

Of note, recently our study was amended to continue the enrollment of RCC patients, then since everolimus is no longer used, with the new cohort of patients we will be able to understand if it could actually have had a negative role by making an internal comparison in the study.

Nevertheless, in our study two of the 3 patients who obtained a PR are still alive and the third had a 7-year OS, predominantly free of treatment. Moreover, the shorter and repeated HD IL-2 schedule proved highly manageable for our Medical Oncology team and did not require the involvement of an ICU. Indeed, the 3 days of low-dose steroids and accurate monitoring of vital signs enabled us to guarantee IL-2 dose density and intensity without apparently compromising the immunological activity of the treatment. Overall, our clinical and immunological results confirm that dexamethasone administration during IL-2 infusion is able to reduce most of the limiting toxicities of the therapy and, with the limits of a non-randomized study, without apparently affecting its efficacy. In this line, from the studies with ICIs and from the first study with Ipilimumab we have learned that corticosteroids even if used at high doses, to treat immune-related toxicity, do not impact the response. At the same time, it is true that patients already undergoing corticosteroids treatment for symptomatic purposes when treated with immunotherapy respond less (33, 34). Notably, all the patients treated in our study were steroid free as required by the inclusion/exclusion criteria.

Although the sample size was too small to identify any significant associations between biomarker changes and clinical outcome, the treatment was immunologically active. Of note, we confirmed immunological responses to specific TAAs by IFN-γ ELISPOT in 16 of the 19 evaluable patients, fulfilling the primary endpoint of the study (**Figure 2**). Interestingly, we detected an increased immunological response in 9/16 patients immediately after the first cycle of the combined therapy (HD IL-2 + RT), while 4 patients required another treatment cycle before obtaining an immunological response against several TAAs. Furthermore, circulating cytokine profiling highlighted a significant increase in IL-8 and IL-10 levels after treatment, mainly in melanoma patients (**Figure 3**). Moreover, comparing baseline and post-treatment values of peripheral blood components, we observed a significant increase in leukocyte and lymphocyte absolute counts after therapy, whereas the neutrophil count remained substantially unchanged (**Figure 4**).

Notably, exploring circulating T cell landscape changes after therapy, we noted a significant decrease in the percentage of CD8+ and CD4+ naïve T cells. Concomitantly, CD4+ effector and effector memory subsets increased, albeit not significantly, after treatment. Although no significant modulations in CD4+ regulatory T cells (Treg) were seen in any patient after therapy, we observed a substantial increase in this cell subset early in the treatment of melanoma patients, and mainly in non-responder ones (**Figure 5**). This suggests that HD IL-2 efficacy may have been limited by the simultaneous promotion of anti-tumor CD8+ T cell and tumor-protective regulatory T cell proliferation. Attempts to overcome these unwanted effects have led to the development of novel compounds targeting subunits of the IL-2 receptor.

Of note, our data show that the combination therapy did not induce a downregulation of tumor marker expression within the tumor. Although an increase in the number of intratumoral CD8+ T cells was observed, the differences observed between pre- and post-treatment biopsies were not significant because of the low number of matched biopsies available.

Regarding the therapy, it must be said that ICIs have found an increasingly anticipated application (up to adjuvant therapy) for both melanoma also in association with targeted therapies, and for RCC with antiangiogenics (45–48). However, these new first line combination therapies and adjuvant applications determine a lack of therapeutic options for progressing patients, so in this scenario HD IL-2 + radiotherapy could play a new role. Moreover, ICIs and IL-2 promote antitumor immunity through different mechanisms and could be employed sequentially or in combination to further boost the immune response, potentially achieving increased efficacy. On this line, combinations of pegylated IL-2 derivatives (e.g. bempegaldesleukin, THOR-707) and immune checkpoint inhibitors are now being investigated in many different settings, including RCC. However, the limited number of patients in this study does not allow to evaluate the impact of previous nivolumab to the efficacy of IL-2.

Our study might share some similarities with the NIVES study, in which the investigators did not meet the primary end point of improving response rate to 40% even if it showed an important DCR and low toxicity (49). Similar to our study, the NIVES study has some limitations e.g. the small sample size, the lack of randomization and the heterogeneity of the patients cohort, but has a different therapy sequence. Indeed, in the NIVES study radiotherapy followed the first infusion of nivolumab. On this point, the authors commented that maybe a different timing of RT and immunotherapy would be better. Probably the synergistic effect between radiotherapy and immunotherapy overcomes the abscopal effect. This synergism might be read as a way of enhancing the systemic effect of immunotherapies, similar to what we could observe with cancer vaccinations followed by immunomodulating drugs such as cytokines or ICIs.

Our study suggests that HD IL-2 and RT could play a significant role in the therapeutic planning of MM and RCC, justifying further efforts to find new combination therapies and biological predictive markers of response. Larger studies with innovative IL-2 engineered fusion proteins are warranted to confirm our findings and to help shed light on other potential markers/immune populations involved in the response and resistance to treatment. In our opinion, these studies should also include non-cutaneous melanoma patients that could have quite an unexpected clinical benefit as our study seems to suggest.

## Data Availability Statement

The raw data supporting the conclusions of this article will be made available by the authors, without undue reservation.

## Ethics Statement 

The studies involving human participants were reviewed and approved by Ethic Committee IRCCS IRST AVR, (approval n° 2217.2012.I.5.81 of 13/06/2012). The patients/participants provided their written informed consent to participate in this study.

## Author Contributions

LR conceived the idea for the study. UG and CL were involved in patient enrollment and patient care. LC, LT, and LR were responsible for patient treatment. CC and PC were involved in patient monitoring during treatment. GG, LV, FR, and MG were responsible for clinical data collection. MP, VA, and GF were responsible for blood sample collection and for sectioning the FFPE tumor samples. JB, CP, AG, and EPa performed the experiments. EPe analyzed the clinical and biomarker data, and performed the statistical analysis. JB, CP, EPe, and LR analyzed and interpreted the data. AlR and FF performed and evaluated the CT scan images. JB, EPe, and CP drafted the manuscript. LR, FR, and MG revised the manuscript for important intellectual content. All authors contributed to the article and approved the submitted version.

## Funding

This work was supported by the Italian Ministry of Health (project no. #RF-2009-1547242). The funders had no role in study design, data collection and analysis, decision to publish, or preparation of the manuscript.

## Conflict of Interest

The authors declare that the research was conducted in the absence of any commercial or financial relationships that could be construed as a potential conflict of interest.

## Publisher’s Note

All claims expressed in this article are solely those of the authors and do not necessarily represent those of their affiliated organizations, or those of the publisher, the editors and the reviewers. Any product that may be evaluated in this article, or claim that may be made by its manufacturer, is not guaranteed or endorsed by the publisher.

## Abbreviations

AEC, 3-amino-9-ethylcarbazole; APC, antigen presenting cell; CI, confidence interval; CR, complete response; CLS, capillary leak syndrome; CTCAE, common terminology criteria for adverse events; CTL, cytotoxic T lymphocyte; DAB, 3,3’-diaminobenzidine; DAMP, damage-associated molecular pattern; DC, dendritic cell; DCR, disease control rate; DMSO, dimethyl sulfoxide; ELISA, enzyme-linked immunosorbent assay; EOT, end of treatment; FSC, forward scatter, FFPE, formalin-fixed paraffin-embedded; HD IL-2, high-dose interleukin-2; ICIs, immune checkpoint inhibitors; ICU, intensive care unit; IHC, immunohistochemistry; irRC, immune-related response criteria; LAK, lymphokine-activated killer; LMR, lymphocyte to monocyte ratio; MHC, major histocompatibility complex; MM, metastatic melanoma; NLR, neutrophil to lymphocyte ratio; OD, optical density; OS; overall survival; PICC, peripherally-inserted central catheter; PBMC, peripheral blood mononuclear cell; PD, progressive disease; PFS, progression free survival; PLR, platelet to lymphocyte ratio; PR, partial response; RECIST, response evaluation criteria in solid tumors; RCC, renal cell carcinoma; RT, radiotherapy; SSC, side scatter; SD, stable disease; SFCs, spot-forming cells; TAA, tumor associated antigens; TME, tumor microenvironment; TKIs, tyrosine kinase inhibitors; Treg, regulatory T cell.
