# Supplementary figures and images for: Radiotherapy and High-Dose Interleukin-2: Clinical and Immunological Results of a Proof of Principle Study in Metastatic Melanoma and Renal Cell Carcinoma
Source: Front Immunol. 2021 Oct 27;12:778459. doi: 10.3389/fimmu.2021.778459 (PMC8578837; doi:10.3389/fimmu.2021.778459)

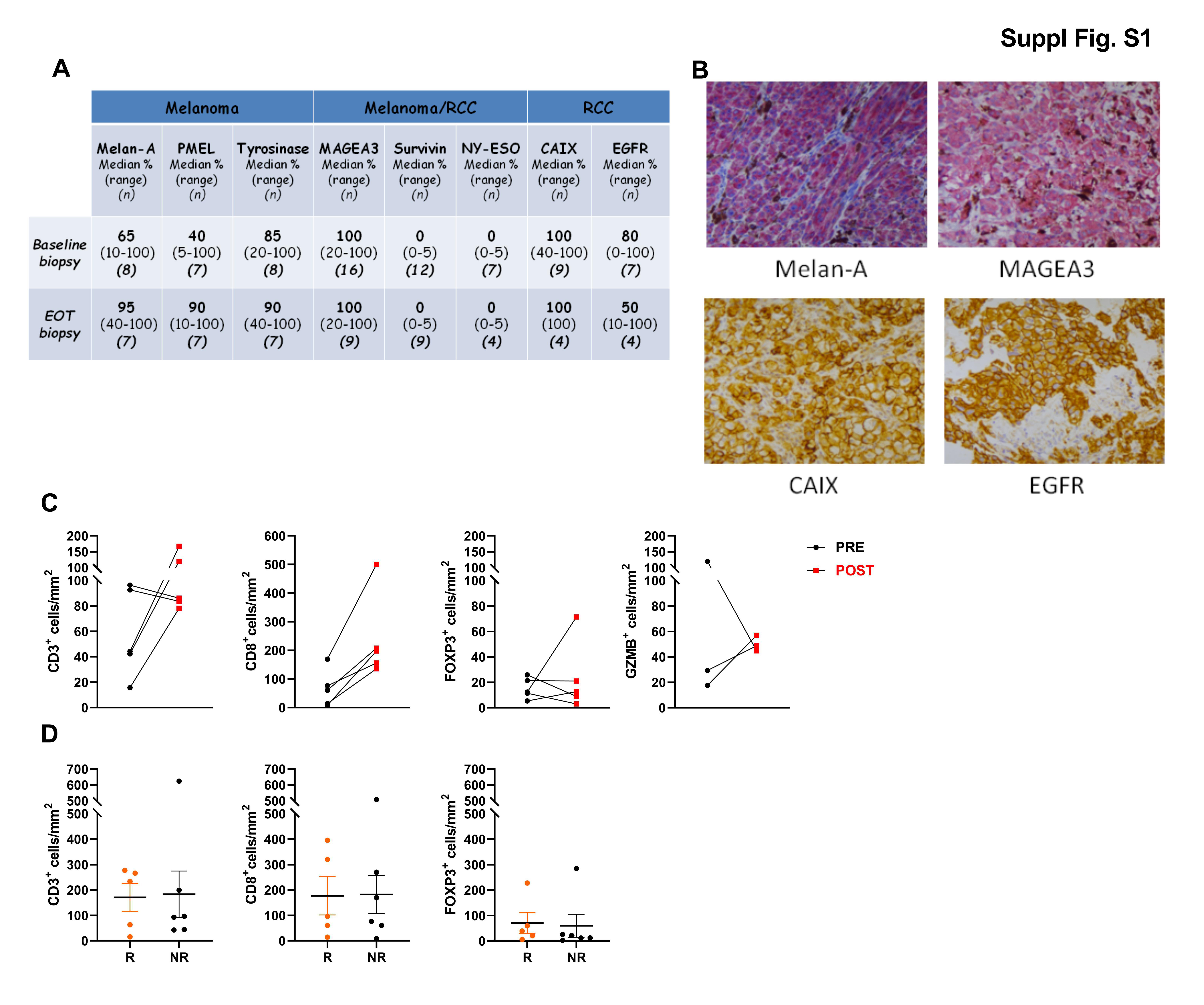

Supplement: Supplementary file 1 [file Image_1.jpeg]
