# Supplementary material for: Radiotherapy and High-Dose Interleukin-2: Clinical and Immunological Results of a Proof of Principle Study in Metastatic Melanoma and Renal Cell Carcinoma
Source: Front Immunol. 2021 Oct 27;12:778459. doi: 10.3389/fimmu.2021.778459 (PMC8578837; doi:10.3389/fimmu.2021.778459)
Supplement: Supplementary file 2 [file Table_1.docx]

**Supplementary Table S1.** Detailed patient characteristics (n=19)

| **Pt#ID** | **Sex** | **Age**^§^ | **Tumor type** | **Site of metastases** | **Previous treatments** | **Cycles of HD IL-2/median dose MIU** | **Tumor site of RT and volume (cc)/total Gy (first,second cycle)** | **BOR (RECIST)/ DoR** | **OS** | **Subsequent treatments** |
| --- | --- | --- | --- | --- | --- | --- | --- | --- | --- | --- |
|  |  |  |  |  |  |  |  |  |  |  |
| ***#001*** | F | 39 | MM | Lung | CT, Ipi, DC vax | 2**/**30.6 | Lung (18.6 cc)/24 Gy,  Lung (19.0 cc)/24 Gy | PD | 12 | CT |
| ***#002*** | M | 39 | RCC | Abdominal lymphnodes | - | 6/35.4 | Lymphnodes (7.4 cc)/18 Gy, Lymphnodes (59.8 cc)/18 Gy | PR/52 | 81 | Sutent, Nivo |
| ***#003*** | F | 40 | MM | lung, soft tissue | CT | 3/25.2 | Liver (314.2 cc)/18 Gy | PD | 5 | FMT |
| ***#004*** | M | 55 | RCC | Lung, bone, lymphnodes, liver | EVE, sorafenib, ldIFN | 6/30.6 | Bone (149.1 cc)/20 Gy,  Lymphnodes (49.8 cc)/24 Gy | SD/6 | 14 | sutent |
| ***#005*** | F | 35 | Uveal MM | Lung, lymphnodes, soft tissue, liver | CT, Ipi | 2/34.2 | Soft tissue (167 cc)/24 Gy,  Lung (62.8 cc)/18 Gy | PD | 2 | - |
| ***#006*** | M | 68 | RCC | Lung, pancreas, lymphnodes, bone | Sutent, EVE, sorafenib | 2/35.4 | Lung (22.8 cc)/18Gy,  Lymphnodes (5.4 cc)/18 Gy | PD | 9 | ldIFN |
| ***#007*** | M | 60 | RCC | Lymphnodes, bone, skin/soft tissue | Sutent, EVE, sorafenib | 6/36.0 | Skin (116.7 cc)/18 Gy,  Lymphnodes (21.13 cc)/18 Gy | PR/9 | 52+ | Nivo |
| ***#008*** | F | 61 | RCC | Lymphnodes, pancreas, lung | Sutent, Nivo, EVE, sorafenib | 6/32.4 | Lymphnodes (7.8 cc)/24 Gy,  Lymphnodes (233.2 cc)/18 Gy | SD/5 | 8 | ldIFN |
| ***#009*** | F | 61 | RCC | Lung, liver, lymphnodes, bone, peritoneum | Pazopanib, EVE, sorafenib | 2/32.0 | Lung (11.6 cc)/18 Gy,  Lung (17.8 cc)/18 Gy | PD | 6 | ldIFN |
| ***#010*** | M | 66 | Uveal MM | Liver, lung, skin, kidney, mesentery | HL-FMT, Ipi, Nivo | 6/36.0 | Liver (73.1 cc)/24 Gy,  Liver (62.5 cc)/24 Gy | SD/6 | 19 | CT |
| ***#011*** | F | 63 | Uveal MM | Liver, lung, lymphnodes | Ipi, Pembro | 6/32.0 | Lymphnodes (7.3 cc)/18 Gy, Lung (3.6 cc)/18 Gy | SD/6 | 7 | - |
| ***#012*** | F | 43 | Uveal MM | Liver, lung, lymphnodes | DTIC, Ipi, Pembro | 6/33.6 | Liver (104.7 cc)/18 Gy | SD/9 | 12 | DC vax |
| ***#013*** | M | 72 | RCC | Lymphnodes, lung, adrenal gland | Sutent, EVE | 4/32.4 | Lymphnodes (20.4 cc)/24 Gy, Lymphnodes (40.2 cc)/18 Gy | SD/2 | 4 | - |
| ***#014*** | M | 52 | RCC | Lung, lymphnodes, bone, peritoneum, adrenal glands | Sutent, sorafenib, EVE | 2/33.0 | Soft tissue (150.2 cc)/24 Gy,  Lung (34.1 cc)/24 Gy | PD | 3 | - |
| ***#015*** | M | 48 | MM | Liver, abdominal lymphnodes | DTIC, Ipi, Nivo | 6/34 | Liver (5.9 cc)/18 Gy,  Lymphnodes (237.2 cc)/18 Gy | PR/21+ | 35+ | - |
| ***#016*** | M | 65 | Uveal MM | Liver, lung, bone, lymphnodes | Ipi, Pembro | 2/33.3 | Lymphnodes (36.4 cc)/18 Gy,  Liver (55.9 cc)/18 Gy | PD | 5 | FMT |
| ***#017*** | F | 40 | RCC (collecting ducts) | Bone, lung, lymphnodes | CT | 1/28 | Lymphnodes (4.4 cc)/18 Gy | PD | 2 |  |
| ***#018*** | F | 72 | Uveal MM | Lung, liver, lymphnodes, skin | Ipi, Pembro, FMT | 4/28.8 | Lymphnodes (23.5 cc)/18 Gy, Lymphnodes (13.0 cc)/18 Gy | PD | 8 | - |
| ***#019*** | M | 68 | Uveal MM | Lung,liver | Ipi, Pembro | 6/32.0 | Liver (365.4 cc)/24 Gy,  Lung (5.7 cc)/18 Gy | SD/4 | 7 | - |

^§^*Patient age is expressed in years, other times are expressed in months. Abbreviations: HD IL-2, high-dose interleukin-2; BOR, best overall response; DoR, Duration of Response; OS, overall survival; MM, metastatic melanoma; RCC, renal cell carcinoma; RT, radiotherapy; Gy, Gray (new international system (SI) unit of radiation dose); EVE, Everolimus; CT, chemotherapy; DC vax, dendritic cell vaccination; DTIC, dacarbazine; FMT, fotemustine; HL-FMT, hepatic locoregional fotemustine infusion; ldIFN, low-dose Interferon; Ipi, Ipilimumab; Pembro, pembrolizumab; Nivo, nivolumab*
