# Supplementary material for: Radiotherapy and High-Dose Interleukin-2: Clinical and Immunological Results of a Proof of Principle Study in Metastatic Melanoma and Renal Cell Carcinoma
Source: Front Immunol. 2021 Oct 27;12:778459. doi: 10.3389/fimmu.2021.778459 (PMC8578837; doi:10.3389/fimmu.2021.778459)
Supplement: Supplementary file 3 [file Table_2.docx]

**Supplementary Table S2.** Details of IHC antibodies

| **Antibody (Ab)** | **Clone** | **Isotype/Host** | **Supplier**  **Cat#** | **Dilution/Ab reaction** | **Antigen retrieval** | **Ab diluent** | **Staining method** |
| --- | --- | --- | --- | --- | --- | --- | --- |
| Melan-A | A103 | Mouse monoclonal | Dako  Cat#M7196 | 1:30/1h RT | Ultra CC1 (Cat#950-224) 100°C, 30 min | Ventana Antibody Diluent  Cat#251-018 | AP RED |
| PMEL | HMB45 | Mouse monoclonal | Dako  Cat#M0634 | 1:50/1h RT | Ultra CC1 (Cat#950-224) 100°C, 30 min | Ventana Antibody Diluent  Cat#251-018 | AP RED |
| Tyrosinase | T311 | Mouse monoclonal | Dako  Cat#M3623 | 1:100/1h RT | Ultra CC1 (Cat#950-224) 100°C, 30 min | Ventana Antibody Diluent  Cat#251-018 | AP RED |
| MAGE-A3 | 1H1 | Mouse monoclonal | Abcam  Cat#ab140678 | 1:150/1h RT | Ultra CC1 (Cat#950-224) 100°C, 60 min | Ventana Antibody Diluent  Cat#251-018 | DAB/AP RED |
| Survivin | 12C4 | Mouse monoclonal | Dako  Cat#M3624 | 1:100/1h RT | Ultra CC1 (Cat#950-224) 100°C, 60 min | Ventana Antibody Diluent  Cat#251-018 | DAB/AP RED |
| NY-ESO1 | 4DB | Mouse monoclonal | Abcam  Cat#ab139339 | 1:150/1h RT | Ultra CC1 (Cat#950-224) 100°C, 60 min | Ventana Antibody Diluent  Cat#251-018 | DAB/AP RED |
| CAIX | / | Rabbit  polyclonal | Abcam  Cat#ab15086 | 1:1000/1h RT | Ultra CC1 (Cat#950-224) 100°C, 60 min | Ventana Antibody Diluent  Cat#251-018 | DAB |
| CD3 | PS1 | IgG2a/Mouse monoclonal | Leica  Cat#NCL-L-CD3-PS1 | 1:150/1h RT | Citrate buffer (Ph6) water bath 98.5°C, 20 min | Ventana Antibody Diluent  Cat#251-018 | DAB |
| CD8 | 4B11 | IgG2b/Mouse monoclonal | Novocastra Cat#NCL-L-CD84B11 | 1:100/1h RT | TRIS EDTA (Ph9) water bath 98.5°C, 20 min | Ventana Antibody Diluent  Cat#251-018 | DAB |
| Foxp3 | SP97 | IgG/Rabbit monoclonal | Thermo Fisher Scientific  Cat#MA5-16365 | 1:100/1h RT | EDTA (Ph8) water bath 100°C, 40 min | PBS+1%BSA | AEC |
|  | 236A/E7 | IgG1/Mouse monoclonal | Abcam  Cat#ab20034 | 1:100/1h RT | Citrate buffer (Ph6) water bath 98.5°C, 20 min | Ventana Antibody Diluent  Cat#251-018 | DAB |
| Granzyme B | GrB-7 | IgG2a/Mouse monoclonal | Merk/Millipore  Cat#MAB3070 | 1:20/1h RT | EDTA (Ph8) water bath 100°C, 40 min | Ventana Antibody Diluent  Cat#251-018 | AEC |
|  | GrB-7 | IgG2a/Mouse monoclonal | Merk/Millipore  Cat#MAB3070 | 1:20/1h RT | Citrate buffer (Ph6) water bath 98.5°C, 20 min | Ventana Antibody Diluent  Cat#251-018 | DAB |

Abbreviations: IHC, Immunohistochemistry; AEC, 3-amino-9-ethylcarbazole; DAB, 3,3'-diaminobenzidine; AP RED, Alkaline Phosphatase Red Detection Kit; TRIS EDTA, ethylenediamine tetraacetic acid; RT, room temperature; h, hour; Ph, potential of hydrogen.
