# Supplementary material for: Radiotherapy and High-Dose Interleukin-2: Clinical and Immunological Results of a Proof of Principle Study in Metastatic Melanoma and Renal Cell Carcinoma
Source: Front Immunol. 2021 Oct 27;12:778459. doi: 10.3389/fimmu.2021.778459 (PMC8578837; doi:10.3389/fimmu.2021.778459)
Supplement: Supplementary file 4 [file Table_3.docx]

**Supplementary Table S3.** Treatment-related toxicities

| **Type of AE** | **All grades** | | **≥ 3 grade** | |
| --- | --- | --- | --- | --- |
|  | **n** | **(%)** | **n** | **(%)** |
| Fever | 13 | (68.4) | 0 | (0.0) |
| Erythema/rash | 11 | (57.9) | 2 | (10.5) |
| Pain | 8 | (42.1) | 2 | (10.5) |
| Diarrhea | 6 | (31.6) | 2 | (10.5) |
| Vomiting | 5 | (26.3) | 0 | (0.0) |
| Pruritus | 5 | (26.3) | 0 | (0.0) |
| Skin | 5 | (26.3) | 0 | (0.0) |
| Nausea | 4 | (21.1) | 1 | (5.3) |
| Anorexia | 3 | (15.8) | 0 | (0.0) |
| Asthenia | 2 | (10.5) | 1 | (5.3) |
| Cough | 1 | (5.3) | 0 | (0.0) |
| Hypotension | 1 | (5.3) | 0 | (0.0) |
| Infection | 1 | (5.3) | 0 | (0.0) |
| Thrombocytopenia | 1 | (5.3) | 0 | (0.0) |
| Oliguria with elevated serum urea and serum creatinine | 1 | (5.3) | 0 | (0.0) |

Abbreviations: AE, adverse event
